# Supplementary material for: Study on Long-Term Tracing of Fibroblasts on Three-Dimensional Tissue Engineering Scaffolds Based on Graphene Quantum Dots
Source: Int J Mol Sci. 2022 Sep 20;23(19):11040. doi: 10.3390/ijms231911040 (PMC9570154; doi:10.3390/ijms231911040)
Supplement: Supplementary file 1 [file ijms-23-11040-s001.zip › ijms-1902192-supplementary.pdf]

The results of fluorescence attenuation of fibroblasts in seven days:

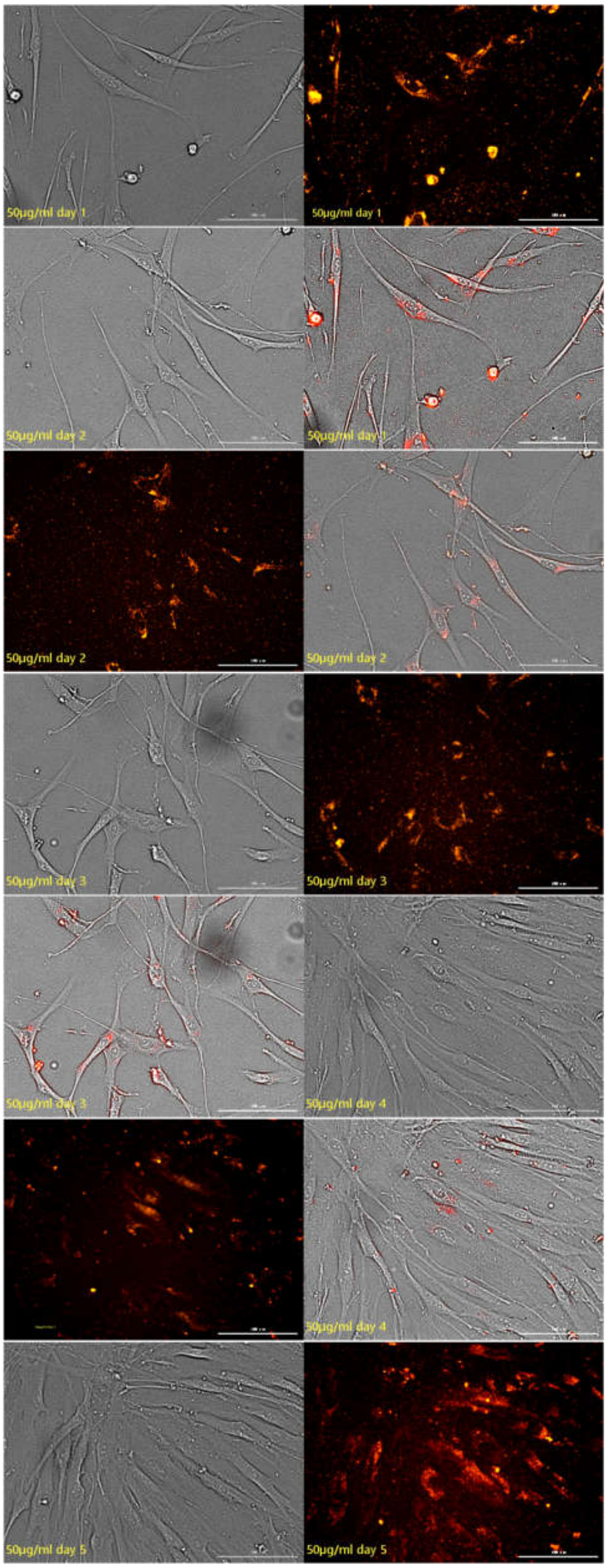

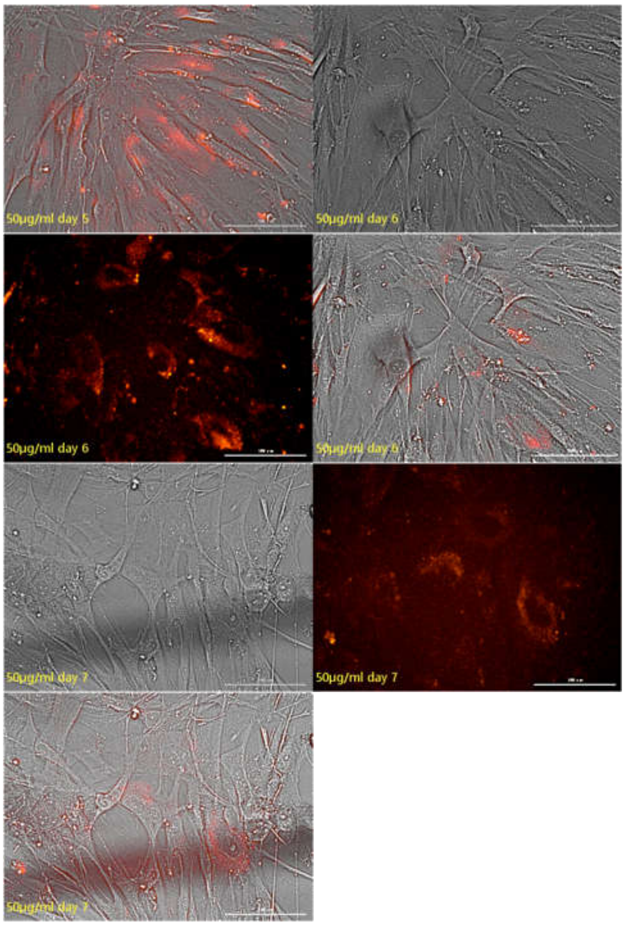

Figure S1. The fluorescence map from the first day to the seventh day, about 50 μg/mL AGQDs-labeled fibroblasts. From left to right, there are bright field, fluorescent field and composite pictures, respectively.

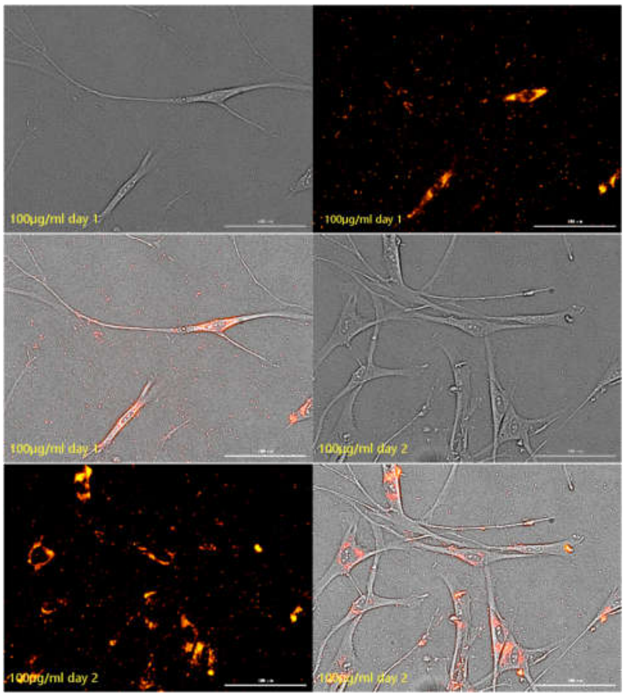

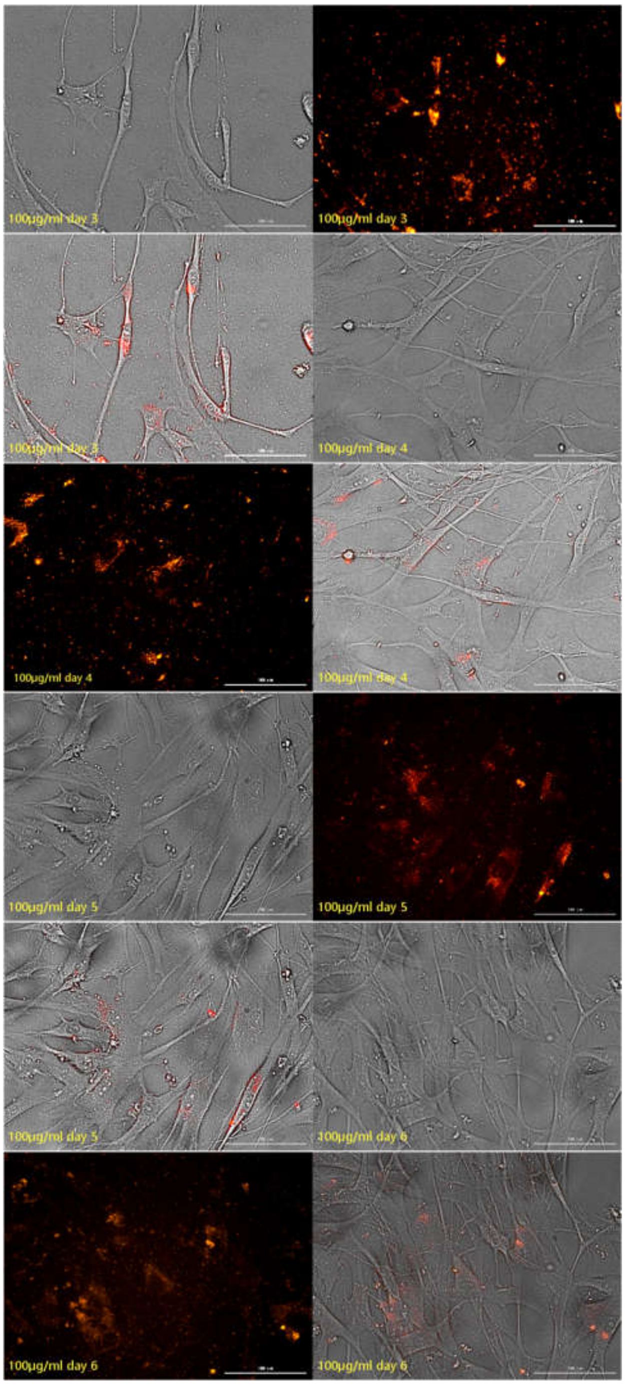
100µg/ml day 3
100µg/ml day 3
100µg/ml day 3
100µg/ml day 4
100µg/ml day 4
100µg/ml day 4
100µg/ml day 5
100µg/ml day 5
100µg/ml day 5
100µg/ml day 6
100µg/ml day 6
100µg/ml day 6

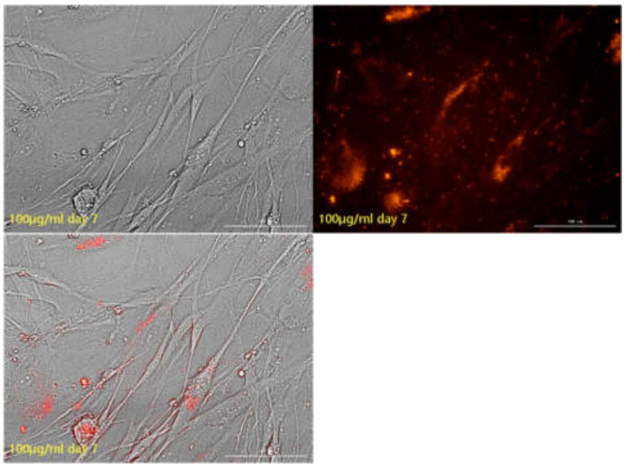

Figure S2. The fluorescence map from the first day to the seventh day, about 100 μg/mL AGQDs- labeled fibroblasts,. From left to right, there are bright field, fluorescent field and composite pictures, respectively.

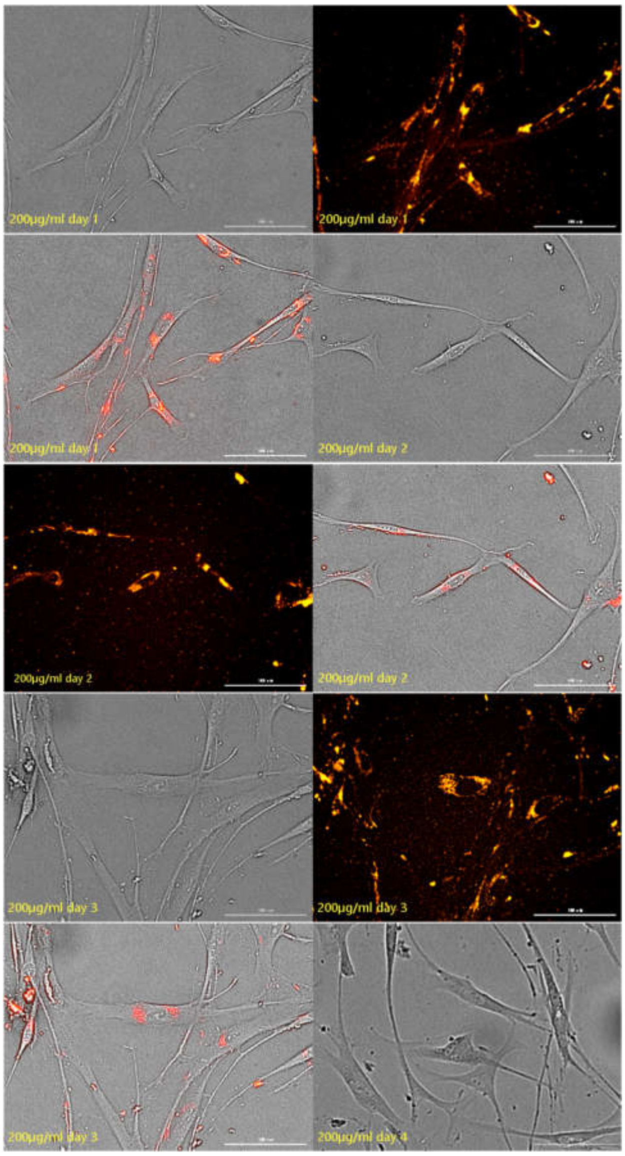

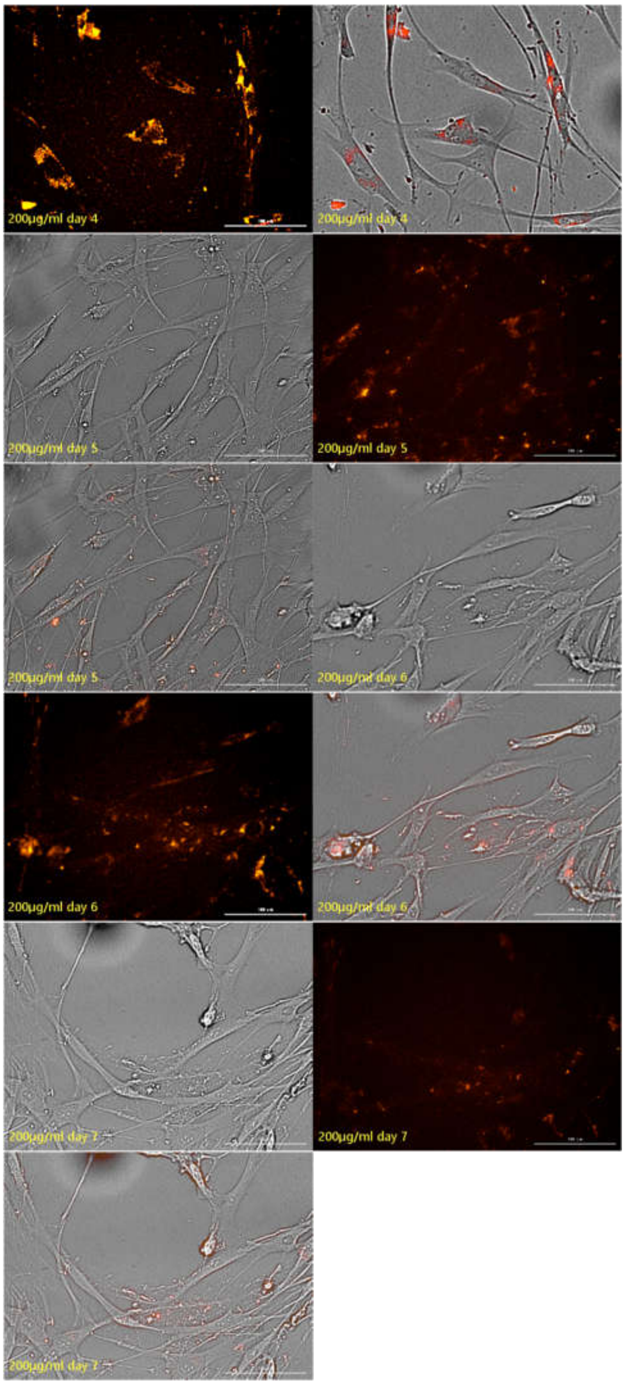

Figure S3. The fluorescence map from the first day to the seventh day, about 200 μg/mL AGQDs- labeled fibroblasts,. From left to right, there are bright field, fluorescent field and composite pictures, respectively.
